# Impact of COVID-19 on pediatric asthma-related healthcare utilization in New York City: a community-based study

**DOI:** 10.1186/s12887-023-03845-1

**Published:** 2023-01-23

**Authors:** Erin Thanik, Kaoru Harada, Elizabeth Garland, Moira Bixby, Jasmine Bhatia, Ray Lopez, Sergio Galvez, Elan Dayanov, Krishna Vemuri, Douglas Bush, Nicholas B. DeFelice

**Affiliations:** 1 Department of Environmental Medicine and Public Health, Icahn School of Medicine at Mount Sinai, One Gustave L. Levy Place, Box 1057, New York, NY 10029 USA; 2 Department of Medicine, Division of Clinical Immunology, Icahn School of Medicine at Mount Sinai, New York, NY USA; 3 LSA Family Health Service, New York, NY USA; 4 Graduate Program in Public Health, Icahn School of Medicine at Mount Sinai, New York, NY USA; 5 Department of Pediatrics, Division of Pulmonary, Icahn School of Medicine at Mount Sinai, New York, NY USA; 6 Department of Global Health, Icahn School of Medicine at Mount Sinai, New York, NY USA

**Keywords:** COVID-19, Asthma, Health disparities, Social determinants of health

## Abstract

**Background:**

COVID-19 disproportionately affects families of low socioeconomic status and may worsen health disparities that existed prior to the pandemic. Asthma is a common chronic disease in children exacerbated by environmental exposures.

**Methods:**

A cross-sectional survey was conducted to understand the impact of the initial stage of the pandemic on environmental and social conditions, along with access to care for children with asthma in New York City (NYC). Participants were recruited from a community-based organization in East Harlem and a nearby academic Pediatric Pulmonary clinic and categorized as having either public or private insurance (*n* = 51).

**Results:**

Factors significantly associated with public compared to private insurance respectively were: increased reports of indoor asthma triggers (cockroach 76% vs 23%; mold 40% vs 12%), reduced income (72% vs 27%), and housing insecurity (32% vs 0%). Participants with public insurance were more likely to experience conditions less conducive to social distancing compared to respondents with private insurance, such as remaining in NYC (92% vs 38%) and using public transportation (44% vs 4%); families with private insurance also had greater access to remote work (81% vs 8%). Families with public insurance were significantly more likely to test positive for SARS-CoV-2 (48% vs 15%) but less likely to have gotten tested (76% vs 100%). Families with public insurance also reported greater challenges accessing office medical care and less access to telehealth, although not statistically significant (44% vs 19%; 68% vs 85%, respectively).

**Conclusions:**

Findings highlight disproportionate burdens of the pandemic, and how these disparities affect children with asthma in urban environments.

**Supplementary Information:**

The online version contains supplementary material available at 10.1186/s12887-023-03845-1.

## Introduction

Shortly after coronavirus disease 2019 (COVID-19) was declared a global pandemic by the World Health Organization in March of 2020, New York City (NYC) became the epicenter of the crisis [1]. As COVID-19 spread across NYC, patterns of health inequities emerged in viral testing, hospitalizations, and deaths. In the initial stages of the pandemic, testing rates by zip-code directly correlated with the proportion of white residents, while hospitalization and death were highest among Black and Hispanic/Latino populations [1, 2]. In NYC, hospitalizations and deaths related to COVID-19 were highest in the Bronx, which is the borough that has the highest proportion of racial/ethnic minorities and lowest household median income; Manhattan, the most affluent borough with a predominantly white population, had the lowest numbers of hospitalizations and deaths [3].

The pandemic also affected health care utilization patterns, and the total number of emergency department (ED) visits in the United States (US) declined by 42% between March 29-April 25,^th^ 2020 compared to the same period in 2019 [4]. The largest decrease was seen in patients ≤ 14 years of age, and among children ≤ 10 years of age there was an 84% reduction in asthma-related ED visits [4]. In NYC, a large medical institution pediatric ED had a 92% reduction in asthma-related admissions during the 12 weeks following the city’s stay-at-home order on March 22^nd^, 2020 relative to the prior year [5].

Asthma is one of the most common chronic medical conditions in children, and pediatric asthma services were significantly impacted by the pandemic as providers reduced in-person visits and accepted fewer new patients [6]. Furthermore, asthma is exceptionally vulnerable to changes associated with the pandemic due to its well-established environmental and viral triggers [7–9]. Stay-at-home orders, especially in urban environments, may lead children of low socioeconomic status to face greater exposure to indoor allergens known to be related to substandard housing conditions, such as cockroach and mold [10], while also having close contact with family members at higher risk of COVID-19 infection [11].

Children in the low-income neighborhood of East Harlem, NYC, are disproportionately affected by asthma morbidity compared to their geographically close neighbors [12]. Residents of East Harlem also suffered a high burden of COVID-19 disease; during the first 6 months of the pandemic the COVID-19 mortality rate in East Harlem was 3.1 times higher compared to a nearby more affluent neighborhood, the Upper East Side [13]. As the disparities in COVID-19 morbidity and mortality became evident, there was a growing concern that chronic disease health disparities that existed prior to the pandemic may also be exacerbated. We conducted a survey in collaboration with a community-based organization (CBO) in East Harlem and a nearby Pediatric Pulmonary clinic in an academic medical center to investigate how the initial stages of the COVID-19 pandemic impacted asthma and COVID-19 care, home environmental conditions, and social determinants of health for children with asthma.

## Methods

### Study population

Participants were recruited from two sites that serve families with children who have asthma: LSA Family Health Services (LSA) and a Pediatric Pulmonary clinic in an academic medical center. These sites are located in geographically close but distinct neighborhoods in the borough of Manhattan, NYC: 1) East Harlem (included zip codes 10029, 10035) and 2) Upper East Side (10021, 10028, 10128, 10065, 10075, and 10162).

**Fig. 1:**
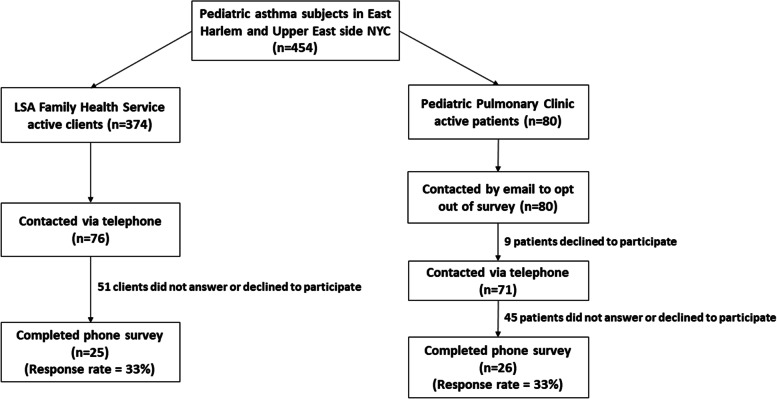
Flowchart of participants recruited from both study sites outlining response rates. Recruitment goal was 25 participants from each site

LSA is a non-profit community-based organization and certified home health agency located in the neighborhood of East Harlem, committed to improving the health of high-risk families in the community since 1958. East Harlem is a high-poverty neighborhood with 23% of residents living in poverty, higher than the rest of Manhattan, with well documented social and health disparities affecting its residents [14]. LSA operates several programs that address the social determinants of health needs of East Harlem residents. We recruited participants who were enrolled in their Environmental Health Services Program, which assists families in addressing indoor asthma triggers. Participants were contacted from a random generated list of 374 active Environmental Health Services Program participants. LSA reached out to 76 clients via telephone, of which 51 did not answer or declined to participate, and 25 clients completed the survey yielding a response rate of 33% (Fig. 1).

Participants were also recruited from a Pediatric Pulmonary clinic located in a large academic hospital located at the border of the East Harlem and Upper East Side neighborhoods. The Upper East Side is a low-poverty neighborhood, with only 7% of its residents reporting incomes below the poverty line, which is 50% less than the rest of Manhattan as a whole [14]. Potential participants were identified by a list of children with asthma who had attended the Pediatric Pulmonary clinic in the past 3 years from the Mount Sinai Data Warehouse who met inclusion criteria. Families were contacted in order from a random generated list of 80 potential participants. We emailed these 80 patients to introduce the study and provide an opt out option, of which 9 declined via email to participate. We then attempted to contact 71 patients via telephone, of which 45 either did not answer or declined participation and 26 clients completed the survey yielding a response rate of 36% (Fig. 1).

Families who met the following criteria were eligible to participate: children treated for asthma between 5–18 years of age at the time of survey administration, the ability to complete a survey over the telephone in English or Spanish, and a primary address in one of the 8 zip codes designated for the neighborhoods of East Harlem or the Upper East Side. This minimum age was chosen due to the challenges of diagnosing asthma in children under 5 years of age [15]. The enrolled child had to meet criteria of active asthma, defined for this study by self-report of physician diagnosed asthma and one of the following: use of daily controller asthma medication use, recent rescue inhaler use (≥ 4 times in prior 2 weeks), or at least 1 emergency department/urgent care visit or hospitalization for asthma in the previous 12 months. If the respondent had more than one child with asthma in the household meeting inclusion criteria, the eldest child was chosen for participation.

### Data collection

Study information was conveyed via email to a randomly selected pool of potential participants, and families were subsequently contacted by telephone and screened for interest and eligibility if they did not opt out after 72 h of email distribution. The study tool was a 68-question telephone survey developed by the research team. The survey was a combination of questions created by our study team as well as questions adapted from existing surveys [16–22];. The survey was designed to assess: 1) asthma and COVID-19 health measures [16–21]; 2) attitudes and behavioral changes related to the COVID-19 pandemic [16]; 3) environmental exposures in the home during COVID-19 pandemic [16, 20, 21]; and 4) social determinant of health (e.g., food insecurity, housing stability, financial needs, and healthcare access including telehealth capabilities) [20–22]. The survey asked the participants to answer questions regarding these measures during the first 6 months of the pandemic (between March 1-August 31, 2020). Please see supplemental information for the full survey [16–22].

The survey was conducted via telephone in English or Spanish per the participants’ choice between February 1^st^ 2021 and June 2^nd^ 2021. Participants who completed the survey were given the option to receive a $40 gift card to a national retail store as compensation for their time. This study was approved by the Institutional Review Board (IRB) of the Icahn School of Medicine at Mount Sinai, New York, New York.

### Statistical analysis

Participants were stratified based on whether the child with asthma’s health coverage was provided by private or public (Medicaid) health insurance. Overall differences between groups were tested for using chi-squared tests of independence, Fisher’s exact tests, or independent T-tests, where appropriate. Statistical significance was determined by *p*-values < 0.05. Statistical analyses were performed in R version 4.0.4 (2021–02-15).

Variables were combined to determine if the participant’s child with asthma experienced barriers to accessing medical care. If the participant responded “yes” to one or more of the following challenges we classified it as *challenges accessing office medical care*: could not get through to the healthcare provider; could not get an appointment with the healthcare provider; the wait at the doctor’s office was too long; the provider’s office was not open or, the participant did not have transportation. Similarly, the same approach was used for understanding barriers to care related to the concern about COVID-19 exposure. They were classified as *concern about COVID-19 exposure* if a participant responded “yes” to one or more of the following factors: participant did not want to take public transportation to go to the doctor or participant was concerned about catching COVID-19 at the clinic/doctor’s office.

## Results

Fifty-one families participated in our survey, of which 25 households had public health insurance and 26 households had private health insurance. Compared to those with private insurance, respondents with public insurance were significantly more likely to report less annual income and educational attainment, and to have Spanish as their primary language (Table 1). All of the private insurance respondents reported an annual household income greater than $40,000 while the vast majority of those with public insurance earned below $40,000. Almost three quarters (72%) of public insurance households reported reduced income during the initial stages of the pandemic with almost half (48%) reporting loss of job, while a fifth of private insurance respondents reported job loss (19%, *p* = 0.03) or reduced income (27%, *p* = 0.002). The two groups did not differ in relationship to mean age or sex of the child with asthma.

**Table 1:** Demographics of families who responded to survey (*n* = 51)

	**Public Insurance** **(*n* = 25)​**	**Private Insurance** **(*n* = 26)​**	*p*-value​*
Primary Language			
English	9 (36%)	0 (0%)	** < 0.001**
Spanish	16 (64%)	26 (100%)	
Household Income(annual)			
< $20,000	13 (52%)	0 (0%)	** < 0.001**
$20,000—$39,999	8 (32%)	0 (0%)	
$40,000—$139,999	2 (8%)	3 (12%)	
> $140,000	0 (0%)	20 (77%)	
Refused	2 (8%)	3 (12%)	
Household Employment			
1 or more caregiver had reduction of work	11 (44%)	6 (23%)	0.1
Loss of job	12 (48%)	5 (19%)	**0.03**
Reduced income	18 (72%)	7 (27%)	**0.002**
Child with Asthma, Age in years (Mean [SD])	12 [4.2]	11.1 [3.6]	0.3
Sex (child with asthma)			
Male	15 (60%)	20 (77%)	0.3
Female	10 (40%)	6 (23%)	
Education completed			
Less than 8^th^ grade	9 (36%)	0 (0%)	** < 0.001**
Some high school/HS degree or GED	10 (40%)	0 (0%)	
Some college/bachelor’s degree	5 (20%)	11 (42%)	
Post graduate degree	0 (0%)	15 (58%)	
Refused	1 (4%)	0 (0%)	

^*^Fisher’s exact test used to test hypothesis

The built environment differed between groups during the initial wave of the pandemic (Table 2). There were significant differences between households with public and private insurance respectively in regards to having the ability to work remotely (8% vs 81%), staying in New York City (92% vs 38%), and living in public housing (28% vs 0%) during the first wave of the COVID-19 pandemic. There were also significant differences in modes of transportation between groups, with those with public insurance being more reliant on public transportation (44% vs 4%) and less likely to own a car (12% vs 81%) when compared to those with private insurance. There were also more people per bedroom in households with public insurance (1.4 vs 1.0).

**Table 2:** Social distancing measures between private and public insurance

	**Public Insurance** **(*n* = 25)​**	**Private Insurance** **(*n* = 26)​**	*p*-value*
1 or more caregiver in house moved to remote work​	2 (8%)​	21 (81%)​	** < 0.001​**
Location of Home​			
Stayed in NYC​	​23 (92%)​	​10 (38%)	** < 0.001​**
Changed Homes ​	1 (4%)​	16 (62%)​	
Reasons for Changing Home​			
Larger/More Social Distancing​	0​ (0%)	11 (42%)​	**​ < 0.001​**
Left NYC ​	0​ (0%)	12 (46%)​	** < 0.001​**
Other​	1 (4%)​	7 (27%)​	**0.05​**
Housing Type			
Public Housing​	7 (28%)​	0​ (0%)	**0.004**​
All Modes of Transportation Used​			
Public Transportation (vs not)​	11 (44%)​	​1 (4%)​	**​ < 0.001​**
Walk/Bike ​	19 (76%)​	15 (58%)​	0.2
Own Car​	3 (12%)​	21 (81%)​	** < 0.001​**
Taxi/Car Service (eg. Uber)​	9 (36%)​	4 (15%)​	0.1
I don’t travel or leave home​	1 (4%)​	4 (15%)​	0.4
Crowding​ (Mean [SD]​)			
How Many People in Home	4.6 [1.8]​	4.6 [2.2]	1.0
Number of Bedrooms​	3.5 [0.83]	4.8 [2.1]	**0.004​**
People/# of bedrooms	1.4 [0.64]	1.01 [0.4]	**0.04**

^*^Fisher’s exact test used to test hypothesis

The public insurance families reported significantly more adverse social and environmental determinants of health (Table 3). There were several differences between groups in regards to environmental exposures known to be associated with asthma control. Public insurance households more frequently reported exposures to cockroach (76% vs 23%) and mold (40% vs 12%), while no significant differences in rodent or household pet exposure were found. The majority of public insurance respondents reported food insecurity concerns, including running out of food during the first wave of the pandemic, while only one private insurance respondent reported sometimes running out of food.

**Table 3:** Social and environmental determinants of health between private and public insurance

	**Public Insurance** **(*n* = 25)​**	**Private Insurance** **(*n* = 26)​**	*p*-value*
Cockroach Sightings in Home	19 (76%)​	6 (23%)​	** < 0.001​**
Evidence of Mice/Rats in Home	3 (12%)​	4 (15%)​	1.0​
Visualized or Smelled Mold/Mildew​	10 (40%)​	3 (12%)​	**0.02​**
Evidence of Water Damage	8 (33%)​	4 (15%)​	0.2​
Furry Pets	11 (44%)​	9 (35%)​	0.2​
Worried about Food Running Out			
Never ​	​6 (24%)​	​25 (96%)​	** < 0.001**​
Sometimes​	15 (60%)​	1 (4%)​	
Often​	4 (16%)​	0​ (0%)	
Food Did Run Out (*n* = 20)			
Never​	​2 (11%)	​0 (0%)	**​ < 0.001**
Sometimes​	15 (79%)	1 (100%)	
Often​	2 (11%)	0​ (0%)	
Worried about Losing Housing	8 (32%)​	0​ (0%)	** < 0.001​**

^*^Fisher’s exact test used to test hypothesis

**Fig. 2:**
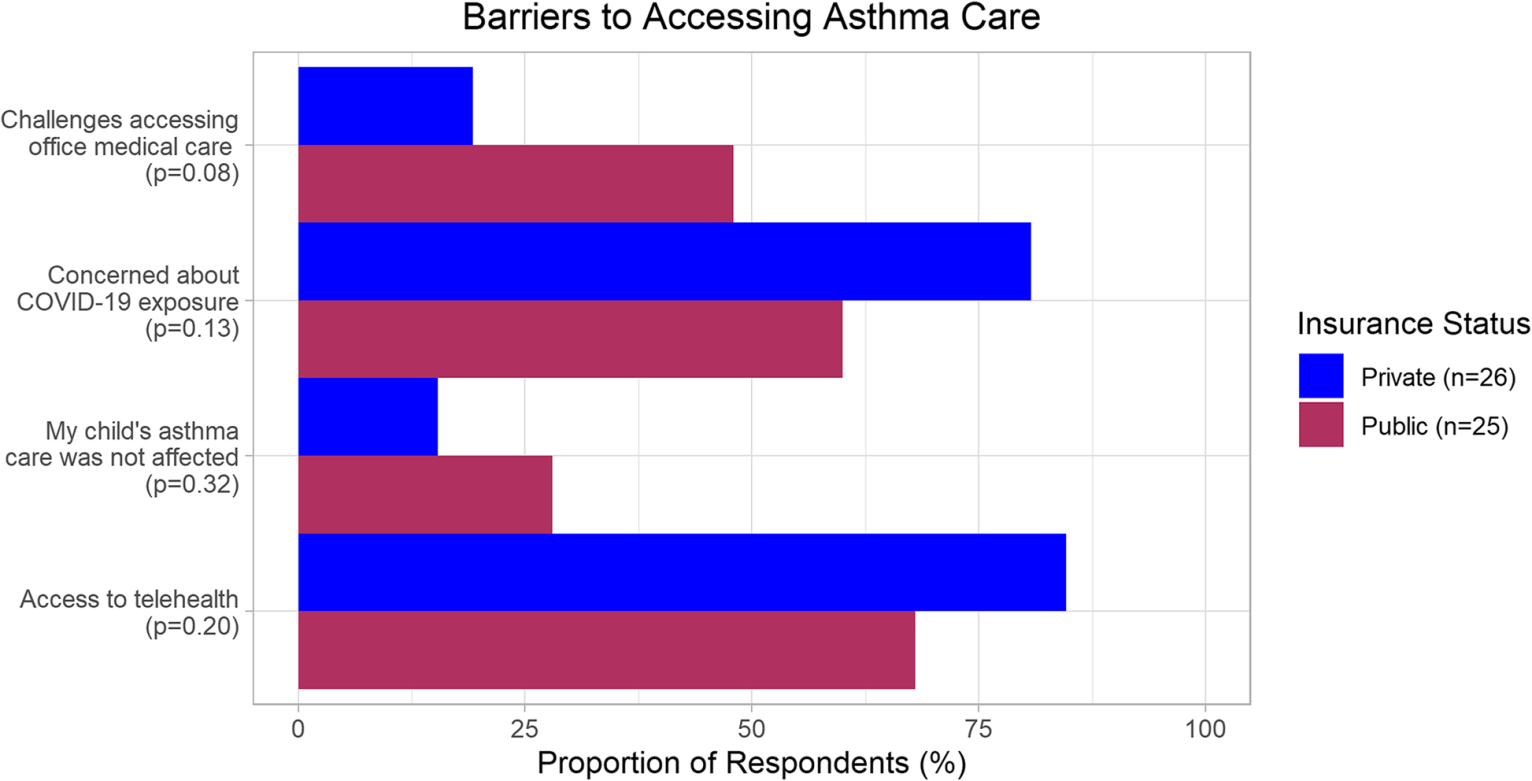
Barriers to accessing asthma care in the first 6 month after COVID-19 pandemic began in New York City, March to September 2020

The differences between both groups regarding accessing medical care were not statistically significant (Fig. 2). Both groups reported that their medical practice offered telehealth, although it was less common among providers of children with asthma with public insurance coverage (68% vs 85%, *p* = 0.2). A greater proportion of public health insurance respondents reported challenges in accessing office medical care compared to respondents covered by private insurance (44% vs 19%, *p* = 0.08). Less than one-third of both groups reported that their child’s asthma care was not affected and more than half of respondents in both groups reported not accessing care due to concerns of COVID-19 exposure.

Three times as many respondents with public insurance reported that a household member was diagnosed with COVID-19 during the first 6 months of the pandemic (48% vs 15%, *p* = 0.02), while more respondents with private insurance reported being tested for COVID-19 during this same time frame (76% vs 100%, *p* = 0.01) (Table 4). There was not a significant difference found in regards to influenza vaccine acceptance with the majority of respondents in both groups planning on receiving influenza vaccination. However, twice as many respondents with private insurance reported planning on receiving COVID-19 vaccination when available (*p* < 0.001).

**Table 4:** Household COVID-19 care and vaccine acceptance between private and public insurance

^**#**^**Household Members includes child with asthma ​**	**Public Insurance** **(*n* = 25)​**	**Private Insurance ​** **(*n* = 26)​**	*p*-value*
Someone in Household^#^ tested positive for SARS-CoV-2	12 (48%) ​	4 (15%)	0.02
Tried to Get Medical Care or Advice about COVID-19​	15 (60%)​	16 (62%)​	1​.0
Number of Households^#^ where someone took a COVID-19 test (yes/no)​			
PCR SARS-CoV-2 test (nasal swab/saliva)​	​19 (76%)​	​26 (100%)​	​**0.01​**
Hospitalized for COVID-19​	2 (8%)​	0​ (0%)	0.5
Flu Vaccination 2020 (plan on receiving/received)​	17 (68%)​	20 (77%)​	0.5
COVID-19 Vaccination (plan on receiving when available)​			
Disagree​	​10 (40%)​	​1 (4%)​	** < 0.001​**
Neutral ​	2 (8%)​	0​ (0%)	
Agree​	10 (40%)​	22 (85%)​	
Don’t know ​	3 (12%)​	0​ (0%)	

^*^Fisher’s exact test used to test hypothesis

## Discussion

In our cross-sectional study, we did not find statistically significant differences between families utilizing public and private insurance in regards to telehealth or office care access during the initial wave of the COVID-19 pandemic. However, families utilizing public insurance reported less access to social distancing measures with greater environmental and social determinants of poor health compared to families with private insurance in the early stage of the COVID-19 pandemic in NYC. Our findings strengthen the already existing evidence that vulnerable communities were disproportionately affected at the beginning of the pandemic and provide additional insights on how these factors may impact children with asthma.

Social distancing was critical to mitigate the spread of COVID-19, especially in the early stages of the pandemic when pharmaceutical interventions were even more limited. After the New York State On PAUSE executive order was put into effect March 22, 2020, the percentage leaving home declined from 80% in February to 42% by April [23]. Our findings suggest that families with public insurance were less able to fully participate in a variety of social distancing measures. Families with private insurance were more likely to leave NYC, transition to remote work, and had fewer people per bedroom. In contrast, the majority of families with public insurance stayed in NYC and were more likely to rely on conditions and resources that made social distancing difficult, such as public transportation and public housing. Our findings are consistent with recently published data on subway use and community mobility in NYC [24–26]. Sy et al. analyzed subway ridership in NYC during March and April 2020 and found that increased subway mobility was associated with lower median income, greater percentage of persons of color, and greater percentage of essential workers; associations did not remain when adjusted for proportion of essential workers, suggesting the disparities are driven by essential work [24]. Kissler et al. studied the relationship between COVID-19 prevalence and mobility patterns between March and May 2020 and found that infection prevalence was lowest in boroughs with the greatest reductions in morning movements out of and evening movements into the borough, which was thought to reflect commuting for work and inability to social distance [25]. A COVID-19 inequity index for NYC based on a composite measure of neighborhood-level disadvantage found that high COVID-19 inequity index neighborhoods had higher subway use after the New York State On PAUSE executive order [26]. These findings provide additional evidence that the most socially disadvantaged are not only at increased risk of COVID-19 infection but also lack the privilege to fully engage in social distancing interventions.

Stay-at-home orders in urban environments may pose a unique challenge to children with asthma from low socioeconomic status. In NYC, students transitioned to remote learning as schools began closing March 15, 2020 [23]. As children spent more time indoors, asthma risk factors that are associated with the home environment may have become more pertinent. Indoor allergens that are associated with increased risk of asthma development and morbidity include mice, cockroach, and mold [8, 9]. These allergens are particularly prevalent in inner-city homes, and lower family income is associated with higher allergen burden [7, 27–30]. In our study, participants with public insurance were more likely to report cockroach and mold in their homes. These findings, combined with the barriers to social distancing described above, highlight the disparities in risk factors related to the home and built environment which became more pronounced during the pandemic. It is important to note that asthma is a multi-factorial disease, and stay-at-home orders decreased other environmental exposures associated with asthma exacerbations, such as other respiratory viruses, which contribute to the majority of exacerbations in children [31, 32]**.** A pediatric hospital in Philadelphia found that the number of positive Rhinovirus cases in patients with asthma decreased following stay-at-home orders, which may have contributed to the decrease in asthma exacerbations and systemic steroid prescriptions observed during this period [33]. Another study conducted in Israel also showed a pattern of decreased pediatric asthma exacerbations during lockdown periods, followed by a peak in asthma exacerbations 2 weeks post-lockdown when the likelihood of being exposed to respiratory viruses increased with relaxation of social distancing policies and children returning to school [34]**.** The significant role viral infections play in asthma exacerbations highlights the importance of all populations being able to participate in social distancing measures and other mitigation strategies to decrease viral transmission.

Adding to the disproportionate impact of this health crisis, families with public insurance were more likely to report adverse social determinants such as employment changes, as well as food and housing insecurity. Respondents with public insurance were more likely to report disadvantages related to employment such as reduction of work hours, loss of job, and decreased income during the first wave of the pandemic. Majority of families with public insurance reported earning less money (72%), while majority of families with private insurance said that their income did not change (53.8%). These results suggest a widening of pre-existing disparities in income, which has been shown to be an independent risk factor for worse asthma outcomes [35]. Families with public insurance were also more likely to report food and housing insecurities, both of which have been associated with increased risk of asthma [36]. The adverse effect of the pandemic on employment was not unique to NYC [37], and social services and resources such as meal programs and shelters that allow for social distancing are vital to support families that qualify for public assistance during this and future public health crises.

We found that a higher proportion of families with public insurance reported challenges accessing office medical care, although the difference was not statistically significant. Both groups reported that the fear of being exposed to SARS-CoV-2 while getting to or at the physician’s office was a barrier to accessing asthma care. Telemedicine was quickly adopted globally in clinics during the pandemic to allow for continued outpatient medical care while social distancing [6]. This clinical modality was offered by the majority of physicians in both groups, although it alone may not be sufficient to meet patients’ needs for chronic care management: more than two-thirds of families with both private and public insurance felt that the pandemic impacted their child’s asthma care.

Although telemedicine has the potential to mitigate barriers in access to care, availability may still be affected by socioeconomic status. A study evaluating pediatric asthma health care utilization in Philadelphia before and after COVID-19 public health measures were enacted in the spring of 2020 found that video telemedicine was rapidly incorporated; video telemedicine was previously not available but subsequently accounted for 61% of all encounters, while outpatient in-person encounters decreased by 87% [33]. However, the majority (70%) of these video visits were conducted with non-Medicaid patients, and the proportion of patients with Medicaid coverage receiving hospital care increased [33]. Thus, further research regarding disparities and barriers in telemedicine access is necessary to guide future healthcare spending and policies aimed at improving health care equity.

Although COVID-19 testing is a vital diagnostic tool and crucial for contact tracing, testing in the US was restricted and difficult to obtain at the beginning of the pandemic. We found that more families with public insurance reported having a household member who tested positive for COVID-19, despite families with private insurance reporting more frequent testing. Our findings are consistent with recently published data on disparities in COVID-19 testing. A study analyzing COVID-19 testing data from the NYC Department of Health and Mental Hygiene between March and April of 2020 found that the ratio of positive tests to total tests significantly decreased with increasing socioeconomic status index score and increasing proportion of white residents [2]. In contrast, the total number of tests administered significantly increased with higher proportion of white residents [2]. Another study analyzed the distribution of testing sites in NYC by race in May 2020 and found that majority white zip codes had the highest number of test sites, even though the test positive rate was lower than predominantly Black and Latino areas [38]. These results suggest that people in lower-socioeconomic status areas have lower ascertainment rates of infection, thus the perceived risk relative to reported transmission in these communities has greater uncertainty due to limited public health monitoring relative to majority white communities.

Notable differences emerged when we asked our participants about their plan on receiving the COVID-19 vaccine when it became available. Majority (85%) of participants with private insurance stated that they would get the vaccine, compared to 40% of those with public insurance. Although opinions about the vaccine may have changed since our data collection, disparities in vaccination rates have been observed in the US. DiRago et al. examined the number of people in US cities, including NYC, who had received at least one dose of a COVID-19 vaccine [39]. The authors found that ZIP codes with higher shares of people of color and low-socioeconomic status individuals had lower vaccination levels and smaller increases in uptake between March and April of 2021, when vaccine eligibility was expanding [39]. Other factors related to vaccine hesitancy include medical mistrust and perception of safety and vaccine novelty [40, 41]. Prior studies that have shown general distrust in vaccination, including influenza vaccination, was associated with COVID-19 vaccine hesitancy [41, 42]. Interestingly, in our study we found that there was no difference in influenza vaccine acceptance between groups, but families with public insurance were significantly less likely to accept the COVID-19 vaccine. Racial and socioeconomic disparities regarding COVID-19 vaccination are well documented [40, 43, 44] and partially rooted in issues surrounding structural racism and medical mistrust [45].

Our study adds to the growing literature highlighting the social determinants of health needs as well as disparities that were exacerbated by the COVID-19 pandemic. Solutions are complex and multifactorial and require both upstream and downstream interventions. CBOs can play a vital role in helping to combat health disparities through several different mechanisms, such as assisting high-risk communities navigate complicated healthcare systems as well as addressing social determinants of health [46]. These organizations have often earned trust in marginalized communities and are thus well positioned to provide services to help address widening health disparities. Community health workers can identify challenges such as risk of job loss, food insecurity, and overcrowded housing. When NYC was the epicenter of the pandemic in the spring of 2020, CBO’s conducted thousands of virtual wellness checks and addressed social determinants of disparities [46]. Community leaders are also in a unique position to address vaccine hesitancy by providing accurate information and assessing barriers in the community. For example, a community-academic-public health partnership in San Francisco utilized a community-centered vaccination strategy to identify and address barriers to vaccination in the Latinx community and successfully administered over 20,000 vaccines; of those who received the vaccine, 98.4% completed both vaccine doses and 90.7% said they were more likely to recommend vaccination to family and friends after their experience [47]. Our community partner, LSA, played an important role in addressing access and health disparities during the pandemic. They collaborated with multiple organizations to provide onsite COVID testing and to connect staff and community members to vaccination appointments. LSA has since received a COVID Disparities Grant from the New York City Department of Health and Mental Hygiene to hire and train additional community health workers in an effort to connect more community members to services that address social determinants of health, including COVID disparities.

Our study has limitations common in cross sectional survey-based studies, such as recall bias. Data assessing changes in income and employment were self-reported, and exposure to allergens such as cockroach was based on family sightings and not quantitatively measured. Despite these limitations and small sample size, this study revealed disparities in care and social determinants of health based on socioeconomic status. We did not find statistically significant barriers to care, although these differences may have been detected with a larger sample size.

## Conclusion

We found that insurance type (public vs. private insurance) was associated with disparities in social and environmental determinants of health, access to care, and ability to social distance during the beginning of the COVID-19 pandemic for children with asthma in NYC. Our study supports the growing evidence that low socioeconomic households faced a disproportionate burden during the pandemic and provides data illustrating how such disparities impact children with asthma. Further studies evaluating barriers to asthma care are needed to inform policies that support high-risk and vulnerable families in future public health crises.

## Supplementary Information

**Additional file 1.**

## Data Availability

Please see supplemental information for the full survey. Data is available on an aggregate level upon request. For data request, please contact nicholas.defelice@mssm.edu.

## Abbreviations

COVID-19: Coronavirus disease 2019
NYC: New York City
ED: Emergency department
US: United States
CBO: Community-based organizations
LSA: LSA Family Health Services
IRB: Institutional Review Board
