# Supplementary material for: Impact of COVID-19 on pediatric asthma-related healthcare utilization in New York City: a community-based study
Source: BMC Pediatr. 2023 Jan 23;23:41. doi: 10.1186/s12887-023-03845-1 (PMC9868511; doi:10.1186/s12887-023-03845-1)
Supplement: Supplementary file 1 — Additional file 1. [file 12887_2023_3845_MOESM1_ESM.pdf]

Asthma/Covid-19 survey

AAA

We are going to ask you a series of questions regarding your experiences during the COVID-19 pandemic. We will be asking questions related to your child's asthma care during this period, as well as factors that may have affected your entire family. You can refuse to answer any question you don't feel comfortable answering. We would like to talk to someone in your household who can answer health questions about your child with asthma. Would you be able to answer these questions? [Interviewer Note: if not, please identify the best person to answer the survey questions and proceed with survey with identified person]

- ○ Real
- ○ Test

Phone Consent for Impact of COVID-19 on Children with Asthma Study

Hello, my name is __________, calling on behalf of Dr. __________ office at Mount Sinai _______.
We are calling to see if you are willing to help us with a research study. The purpose of this research study is to see how the SARS-CoV-2 outbreak has impacted your child's asthma and is affecting you and your family. You are being asked to take part in a research study if you have a child who is 5-17 years old, with asthma and one of the following: daily controller asthma medication use, recent rescue inhaler use or at least 1 ED/urgent care visit or hospital admission for asthma in the last year. Do you have a few minutes to talk?
If No.
If they don't have time to talk: Would you like me to call you back? When is a good time?
OR
If they do not want to participate: Thank you for your time, I hope you and your family are staying safe! You can always call your doctor's office if you have any questions about your health during this time.
If Yes.
Being in a research study is completely voluntary. You can choose not to be in this research study. You can also say yes now, and change your mind later. Deciding not to be in the research study, now or later, will not affect your ability to receive medical care at Mount Sinai Medical Center.
If you agree to take part in this research, you will be asked to complete the phone survey. Your participation in this study will take about 30-minutes and we will offer a $40 gift card for your time. We expect that 50 people will take part in this research study.
You can choose not to answer any question you do not wish to answer. You can also choose to stop taking the survey at any time. You must be at least 18 years old to participate. If you are younger than 18 years old, you will not be able to answer the survey.
The possible risks to you in taking part in this research are:
Potential loss of confidentiality of data
The possible benefits to you for taking part in this research are:
Our community having a better understanding of the impact the outbreak has had on non-COVID-19 medical care
$40 gift card

To protect your identity as a research subject, no identifiable information will be collected, the research data will not be stored with your name, and the researcher(s) will not share your information with anyone. In any publication about this research, your name or other private information will not be used.
If you have any questions about this research, please contact the researcher at nicholas.defelice@mssm.edu or erin.thanik@mssm.edu. You can also call the Program for the Protection of Human Subjects Office at 212-824-8200.

If Yes.
Thank you for consenting to participate with our survey. You may refuse to answer any questions and can stop

the survey at any time. Your participation will help us better understand the challenges families faced during the pandemic. We appreciate your time.
Continue to survey.

Do you consent to this survey?

- Yes
- No

Completion Date Today

FECHA DE FINALIZACIÓN:

* must provide value

INTERVIEWER's INITIALS

INICIALES DEL ENTREVISTADOR:

* must provide value

Language

IDIOMA:

- English
- Spanish
  Español

We are going to ask you a series of questions regarding your experiences during the COVID-19 pandemic. We will be asking questions related to your child's asthma care during this period, as well as factors that may have affected your entire family. You can refuse to answer any question you don't feel comfortable answering. We would like to talk to someone in your household who can answer health questions about your child with asthma. Would you be able to answer these questions? [Interviewer Note: if not, please identify the best person to answer the survey questions and proceed with survey with identified person]

Vamos a hacerle una serie de preguntas con respecto a sus experiencias durante la pandemia COVID-19. Haremos preguntas relacionadas con el cuidado del asma de su hijo/a durante este período, así como los factores que pueden haber afectado a toda su familia. Puedes negarte a responder a cualquier pregunta que no te sientas cómodo respondiendo. Nos gustaría hablar con alguien en su hogar que pueda responder preguntas de salud sobre su hijo/a con asma. ¿Sería capaz de responder a estas preguntas? [Nota del entrevistador: si no, por favor identifique a la mejor persona para responder a las preguntas de la encuesta y proceder con la encuesta con la persona identificada]

SECTION A: ASTHMA SCREENING QUESTIONS

1.What is the zip code of your home in New York City?

SECCIÓN A: PREGUNTAS DE DETECCIÓN DEL ASMA

1.¿Cuál es el código postal de su casa en la ciudad de Nueva York?

- 10021
- 10035
- 10028
- 10029
- 10129
- 10075
- 10065
- 10162
- If other Zipcode then not eligible for

study
Si es otro código postal, entonces no es elegible para estudiar

- ◯ 10128

(Interviewer Note: Inclusion criteria: zip codes 10029, 10035, 10028, 10128, 10021,10075,10065,10162}

I'd like to ask questions about your children with asthma.

Me gustaría hacer preguntas sobre sus hijo/a con asma.

2.How many of your children who live in your home do you think has asthma?

2.¿Cuántos de sus hijos/as que viven en su hogar cree que tienen asma?

What are their first name/names?

Cuáles son sus nombres?

Expand

How old are they (in years)?

Cuántos años tienen?

[Interviewer Note: List all children with asthma, and first ask questions about the child who was identified for this survey. If there wasn't a child identified for the survey or if the identified child does not meet inclusion criteria, ask survey questions 3-8, starting with the oldest child with asthma and continue with subsequent younger children until you identify a child who meets inclusion criteria]

[Nota del entrevistador: Enumere a todos los niños con asma y primero haga preguntas sobre el niño que fue identificado para esta encuesta. Si no se identificó a un niño para la encuesta o si el niño identificado no cumple con los criterios de inclusión, haga preguntas de la encuesta 3-8, comenzando con el niño mayor con asma y continúe con los niños más pequeños subsiguientes hasta que identifique a un niño que cumpla con los criterios de inclusión]

I'd first like to ask some health questions about {identified child with asthma}

3. What is your relationship to {child with asthma}?

En primer lugar, me gustaría hacer algunas preguntas de salud sobre el niño identificado con asma

Cuál es tu relación con el niño con asma?

- ◯ Mother (mark step if applicable)
  Madre (marque el paso si corresponde)
- ◯ Father (mark step is applicable)
  Padre (se aplica el paso de marca)
- ◯ Brother/Sister (mark step/half if applicable)
  Hermano/Hermana (marque el paso/mitad si corresponde)
- ◯ Grandparent (mark father/mother)
  Abuelo (marca padre/madre)
- ◯ Other Relative

Otro pariente

- Unrelated
  No relacionado
- Don't Know
  No sé
- Refused to answer
  Se nego a responder

[Interviewer Note: Parent/guardian needs to answer affirmatively to question 4 and at least one question between 5-8 to be eligible and to identify active asthma]

[Nota del entrevistador: El padre/guardián debe responder afirmativamente a la pregunta 4 y al menos una pregunta entre 5-8 para ser elegible e identificar el asma activo]

4.Has a doctor, nurse or other health professional ever said that {the child} has asthma?

Alguna vez un médico, enfermera u otro profesional de la salud ha dicho que el niño tiene asma?

- Yes,
  Si
- No [If no, explain that patient is not
  eligible, ask questions 3-8 for next oldest child with reported asthma],
  - No [Si no, explique que el paciente no es elegible, haga preguntas 3-8 para el próximo niño mayor con asma reportada]
- Refused to answer
  Se negó a responder

5. In the past 3 months, what prescription asthma medications did {child with asthma} take by inhaler? [Interviewer Note: See drop down menu including meds and check all that apply, affirmative if at least one medication from list is identified excluding quick relief medications (ventolin, albuterol or proventil)]

En los últimos 3 meses, ¿qué medicamentos recetados para el asma tomo su hijo/a con asma por inhalador? [Nota del entrevistador: Ver menú desplegable incluyendo medicamentos y comprobar todos los que se aplican, afirmativo si al menos un medicamento de la lista se identifica excluyendo los medicamentos de alivio rápido (ventolin, albuterol o proventil)]

- Advair (+ A. Diskus)
- Aerobid
- Albuterol (+ A. sulfate or salbutamol)
- Alupent
- Asmanex (twisthaler)
- Atrovent
- Azmacort
- Beclomethasone dipropionate
- Beclovent
- Bitolterol
- Brethaire
- Budesonide
- Combivent
- Cromolyn
- Flovent
- Flovent Rotadisk
- Flunisolide
- Fluticasone
- Foradil
- Formoterol

- Intal
- Ipratropium Bromide
- Levalbuterol tartrate
- Maxair
- Metaproteronol
- Mometasone furoate
- Nedocromil
- Pirbuterol
- Pro-Air HFA
- Proventil
- Pulmicort Turbuhaler
- QVAR
- Salbutamol (or Albuterol)
- Salmeterol
- Serevent
- Singulair
- Symbicort
- Terbutaline (+ T. sulfate)
- Tilade
- Tornalate
- Triamcinolone acetonide
- V anceril
- Ventolin
- Xopenex HFA
- Other, Please Specify
- no prescription inhalor
- do not know
- refused

Comments/Notes

Comentarios / Notas

Expand

6. During the past 3 months, has {child's name} used the kind of PRESCRIPTION asthma inhaler that gives QUICK relief from asthma symptoms during an attack?[Interviewer Note - this includes medications such as ventolin, albuterol, proventil, or inhaler containing a long acting beta agonist]

Durante los últimos 3 meses, ¿ha utilizado {el nombre del niño} el tipo de inhalador de asma RECETADO que proporciona alivio rápido de los síntomas del asma

- Yes
  Sí
- No
  No
- Don't Know
  No sé
- Refused to answer

durante un ataque? [Nota del entrevistador - esto incluye medicamentos como ventolin, albuterol o proventil or inhaler containing a long acting beta agonist]

Se negó a responder

↺

Now I am going to ask about {your child's} asthma over the past year:

Ahora voy a preguntar sobre el asma de su hijo/a durante el año pasado:

7. During the past 12 months, how many times did {he/she/they} visit an emergency room or urgent care center because of {his/her/their} asthma?

Durante los últimos 12 meses, ¿cuántas veces su niño/a visitó una sala de emergencias o un centro de atención de urgencia debido al asma?

- Times (affirmative if answers one or more times)
  Veces (afirmativa si responde 1 o más veces]
- Zero
  Cero
- Don't Know
  No se
- Refused to answer
  Se negó a responder

↺

Comments/ Notes

Expand

8 **8. During the past 12 months, how many different times did {he/she/they} stay in any hospital overnight or longer because of {his/her/their} asthma?**

- ____Times (affirmative if answers 1 or more times)
- Zero
- Don't Know
- Refused to answer

↺

[Interviewer Note - if answered affirmative to question 4 and at least one of questions 5-8, continue survey. If they did not answer affirmatively to any of questions 3-8, state: I'm sorry, {child with asthma} is not eligible for the survey. Thank you for taking the time to answer our questions." End phone visit or ask questions 3-8 for other children in the home with asthma, if applicable. State: "I'd like to ask questions about your next oldest child with asthma" and then repeat questions 3-8 until you identify a child with asthma who fits criteria for the study]

[Nota del entrevistador - si se responde afirmativamente a la pregunta 4 y al menos una de las preguntas 5-8, continúe la encuesta. Si no respondieron afirmativamente a cualquiera de las preguntas 3-8, Responda: Lo siento, el niño con asma no es elegible para la encuesta. Gracias por tomar el momento de responder a nuestras preguntas. Fin de la visita telefónica o haga preguntas 3-8 para otros niños en el hogar con asma, si corresponde. Estado: "Me gustaría hacer preguntas sobre su próximo hijo/a mayor con asma" y luego repetir las preguntas 3-8 hasta que identifique a un niño con asma que se ajuste a los criterios para el estudio]

Does the child qualify for the survey?

- Yes
- No

SECTION B: ASTHMA CARE DURING PANDEMIC

Now I'm going to ask similar health questions regarding the recent time during the pandemic. The following questions are about medical care for your child's asthma for the 6 months following the first reported case of COVID in New York City (that is, from March 1st until August 31st):

SECCIÓN B: CUIDADO DEL ASMA DURANTE LA PANDEMIA

Ahora voy a hacer preguntas de salud similares con respecto al tiempo reciente durante la pandemia. Las siguientes preguntas son acerca de la atención médica para el asma de su hijo/a durante los 6 meses siguientes al primer caso reportado de COVID en la ciudad de Nueva York (es decir, desde el 1 de marzo hasta el 31 de agosto):

9. During this time period, that is from March 1st until August 31st, did {your child with asthma} have to stay overnight in the hospital because of asthma?

Durante este período de tiempo, es decir, desde el 1 de marzo hasta el 31 de agosto, ¿tuvo que pasar la noche en el hospital debido al asma?

[Interviewer note: this question to capture hospital admissions, not just staying the the ER overnight. There is a separate question for ER visits.]

- Yes
  Sí
- No
  No
- Don't Know/Not Sure
  No sé/No estoy seguro
- Refused to answer
  Se negó a responder

10. Not counting hospitalizations, during this same time period, did {your child with asthma}, go to an emergency room because of asthma?

Sin contar las hospitalizaciones, durante este mismo período de tiempo, ¿su hijo/a con asma,fue a una sala de emergencias debido al asma?

- Yes
  Sí
- No
  No
- Don't Know/Not Sure
  No sé/No estoy seguro
- Refused to answer
  Se negó a responder

11.Not counting hospitalizations or emergency room visits, during this time period, did {your child with asthma} see a doctor or health care provider in the office or clinic either in-person or via Telehealth for asthma?

Sin contar las hospitalizaciones o las visitas a la sala de emergencias, durante este período de tiempo, ¿su hijo/a con asma vio a un médico o proveedor de atención médica en el consultorio o la clínica, ya sea en persona o por telesalud para el asma?

- Yes
  Sí
- No
  No
- Don't Know
  No sé
- Refused to answer
  Se negó a responder

11.2 How many were for a "wheezy episode"? __________

Cuántos fueron para un "episodio de sibilancias en pecho"?

12. During this time period, on how many separate occasions did {your child with asthma} have to take steroid medicine pills or liquid medications for several days because their asthma got worse?

Durante este período de tiempo, ¿en cuántas ocasiones separadas tuvo que tomar píldoras de medicamentos esteroides durante varios días porque su asma empeoró?

- Yes
  Sí
- No
  No
- Don't Know/Not Sure
  No sé/No estoy seguro
- Refused to answer
  Se negó a responder

13. Do you think COVID-19 has impacted your child's asthma care?

Cree que COVID-19 ha impactado el cuidado del asma de su hijo/a?

- Yes
  Sí
- No
  No
- Don't Know
  No sé
- Refused to answer
  Se negó a responder

[BRANCHING]: 13.1 If YES to question 13, ask the following:

How so?

En caso afirmativo a la pregunta 13, pregunte lo siguiente:

Cómo?

Expand

14. Before the pandemic, when you needed medical care for {your child's asthma} right away, how often did you get care as soon as you needed it?

Antes de la pandemia, cuando necesitaba atención médica para el asma de su hijo/a de inmediato, ¿con qué frecuencia recibió atención tan pronto como la necesitó?

- Never
  Nunca
- Sometimes
  Aveces
- Usually
  Por lo general
- Always
  Always
- Don't Know
  No sé
- Refused to Answer
  Se negó a responder

15. Since the COVID-19 pandemic started, how has medical care for {your child's} asthma been impacted?

- Couldn't get through to healthcare provider on the telephone

I will read you a list of options and let me know if you agree with any of the following statements. You can pick more than one option.

[Note to interviewer: mark all that apply]

Desde que comenzó la pandemia COVID-19, ¿cómo se ha visto afectada la atención médica para el asma de su hijo/a?

Le leeré una lista de opciones y me avisa si está de acuerdo con cualquiera de las siguientes declaraciones. Puede elegir más de una opción.

[Nota para el entrevistador: marque todo lo que corresponda]

No pudo comunicarse por teléfono con el proveedor de atención médica
- ☐ Couldn't get an appointment with healthcare provider
  No pudo conseguir una cita con el proveedor de atención médica
- ☐ The wait at the doctor's office was too long once you arrived
  La espera en el consultorio del médico fue demasiado larga una vez que llegó
- ☐ Concerned about catching COVID-19 at the clinic/doctor's office
  Preocupado por contagiarse de COVID-19 en el consultorio de la clínica/médico
- ☐ The clinic/doctor's office wasn't open at a time when you could get there
  El consultorio de la clínica/médico no estaba abierto en un momento en que pudiera llegar
- ☐ Didn't have transportation
  No tenía transporte
- ☐ Didn't want to take public transportation to go to the doctor
  No quería tomar el transporte público para ir al médico
- ☐ The cost of the appointment was too high
  El costo de la cita era demasiado alto
- ☐ My child's asthma care was not affected
  La atención del asma de mi hijo/a no se vio afectada
- ☐ Are there other things we didn't mention that you would like to share? (specify)
  Hay otras cosas que no mencionamos que te gustaría compartir? (especificar):
- ☐ Refused to answer
  Se negó a responder

16. Have you been able to get {your child's} asthma prescription medicines and medical supplies since the start of the pandemic?

Ha podido obtener medicamentos recetados para el asma y suministros médicos de su hijo/a desde el inicio de la pandemia?

- ○ Yes
  Sí
- ○ No
  No
- ○ Don't Know/Not Sure
  No sé/No estoy seguro
- ○ Refused to answer
  Se negó a responder

↺

17. A spacer is a small attachment for an inhaler that makes it easier to use. Does {he/she/they} use a spacer with {his/her/their} asthma medications?

- ○ Yes
- ○ No
- ○ Medication is a dry powder inhaler or

Un espaciador es un pequeño accesorio para un inhalador que hace que sea más fácil de usar. ¿Usa un espaciador con los medicamentos para el asma?

disk inhaler not a canister inhaler
- (Medication has a built-in spacer/does not need a spacer
- Don't know
- Refused to answer

18.Since the COVID-19 pandemic started, did any of {child with asthma}'s health care providers offer Telehealth visits?

[Interviewer note if needed: Telehealth refers to medical visits done over the telephone or video]

Desde que comenzó la pandemia COVID-19, ¿alguno de los proveedores de atención médica de su "niño/a con asma" ofreció visitas de telesalud?

[Nota del entrevistador si es necesario: telesalud se refiere a las visitas médicas realizadas por teléfono o video]

- Yes
  Sí
- No
  No
- Don't Know
  No sé
- Refused to answer
  Se negó a responder

How many Telehealth appointments for {child}'s asthma did you have during this time period?

Cuántas citas de telesalud para el asma de su niño/a tuvieron durante este período de tiempo

SECTION C: COVID CARE FOR CHILD, HOUSEHOLD AND SOCIAL CIRCLE

Thank you for answering questions about your child's asthma. Now we are going to ask questions about {your child with asthma} and your family's health during the COVID-19 pandemic.

Questions about child:

SECCIÓN C: CUIDADO COVID PARA EL hijo/a, EL HOGAR Y EL CÍRCULO SOCIAL

Gracias por responder preguntas sobre el asma de su hijo/a. Ahora vamos a hacer preguntas sobre su hijo/a con asma y la salud de su familia durante la pandemia COVID-19.

Preguntas sobre el niño/a:

19. Since COVID-19 came to New York City (around March), has {your child with asthma} had fever, chills, cough, shortness of breath, muscle aches, diarrhea, or loss of smell or taste?

Desde que COVID-19 llegó a la ciudad de Nueva York (alrededor de marzo), ¿ha tenido su hijo/a con asma fiebre, escalofríos, tos, dificultad para respirar, dolores musculares, diarrea o pérdida de olor o sabor?

- Yes
  Sí
- No
  No
- Don't Know
  No sé
- Refused to answer
  Se negó a responder

20. Have you tried to get medical care or advice from

a doctor or nurse about COVID-19 for {your child with asthma} since the start of the pandemic?

Ha tratado de obtener atención médica o consejo de un médico o enfermera sobre COVID-19 para su hijo/a con asma desde el inicio de la pandemia?

- ◯ Yes
  Sí
- ◯ No
  No
- ◯ Don't Know
  No sé
- ◯ Refused to answer
  Se negó a responder

↺

21. Has {your child with asthma} been tested for COVID-19? The test is a special nose or throat swab, a saliva test or a blood antibody test.

Le han hecho la prueba de Covid19 a su niño/a? La prueba es tomar una pequeña muestra de la nariz o garganta, o de saliva o una prueba de anticuerpos en sangre.

- ☐ Yes
  Si
- ☐ Nose or throat swab was done (if yes proceed to 21.2)
  Nariz o garganta (responde Si, pase a 21.2)
- ☐ Saliva test was done (if yes proceed to 21.3)
  Prueba en saliva(responde Si; pase a 21.3)
- ☐ Blood antibody test was done (if yes proceed to 21.4)
  Anticuerpos en sangre (responde Si; pase a 21.4
- ☐ No (if no proceed to 21.1)
  No (responde No; continua 21.1)
- ☐ Don't know
  No sabe
- ☐ Refused to answer
  Se negó a responder

Questions about household:

We are now going to ask questions about your household during the pandemic. Your household refers to the home you and {child with asthma} stayed in most nights from March 2020-August 2020.

Preguntas sobre el hogar:

Ahora vamos a hacer preguntas sobre su hogar durante la pandemia. Su hogar se refiere al hogar en el que usted y {niño/a con asma} se quedaron la mayoría de las noches desde marzo del 2020 hasta agosto del 2020.

22. How many people, children and adults including you, stayed in that home most nights? (Note you can also include 'refused to answer')

¿Cuántas personas, niños y adultos, incluyendo a usted, se quedaron en esa casa la mayoría de las noches? _______ [menú desplegable, 2-20, se negó a responder]

[ ]

23 Since COVID-19 came to New York City (around March), has anyone in your household, (not including {your child with asthma}) had fever, chills, cough, shortness of breath, muscle aches, diarrhea, or loss of

- ◯ Yes
  Sí
- ◯ No

smell or taste?

Desde que el COVID-19 llegó a la ciudad de Nueva York (alrededor de marzo), ¿alguien en su hogar (sin incluir {su hijo/a con asma}) ha tenido fiebre, escalofríos, tos, dificultad para respirar, dolores musculares, diarrea o pérdida de olor o sabor?

No
- Refused to answer
  Se negó a responder

24. Do you think anyone in your household has or had COVID-19 (not including {your child with asthma})?

Cree que alguien en su hogar tiene o ha tenido COVID-19 (sin incluir {su hijo/a con asma})?

- if yes, how many people in your household?
  Sí, en caso afirmativo, ¿cuántas personas en su hogar?
- No/Not sure
  No / No estoy seguro
- Don't answer
  No respuesta
- Refused to answer
  Se negó a responder

[BRANCHING] 24.1 If YES:

How many?

En caso afirmativo:

¿Cuántos?

25. Have you tried to get medical care or advice for anyone in your household from a doctor or nurse about COVID-19 since the start of the pandemic?

Ha tratado de obtener atención médica o consejos para alguien en su hogar de un médico o enfermero sobre el COVID-19 desde el comienzo de la pandemia?

- Yes
  Sí
- No
  No
- Refused to answer
  Se negó a responder

26. Has anyone in your household been tested for COVID-19 (excluding {your child with asthma})?
The test is a special nose or throat swab, saliva test or a blood antibody test.

(excluyendo su hijo/a con asma)¿Alguien en su hogar se ha hecho la prueba de COVID-19?

La prueba consiste en un hisopo especial de nariz o garganta, una prueba de saliva o una prueba de anticuerpos en sangre.

- Nose or throat swab was done: how many____ (if yes proceed to 27.2)
  Sí, en caso afirmativo, ¿cuántas personas se hicieron la prueba
- Saliva test was done: how many____ (if yes proceed to 27.3)
  Se hizo un frotis de la nariz o la garganta: cuántos ____ (en caso afirmativo, proceda a 27.2)
- Blood antibody test was done: how many____ (if yes proceed to 27.4)
  Se realizó una prueba de saliva: cuántos ____ (si es así, proceda a 27.3)
- No (if no proceed to 27.1)
  Se realizó una prueba de anticuerpos en sangre: cuántos ____ (en caso afirmativo, proceda a

- [ ] Don't know
  No se
- [ ] Refused to answer
  Se negó a responder

28. Was anybody in your household hospitalized for COVID-19?

Alguien de su hogar fue hospitalizado por COVID-19?

- ( ) Yes
  Sí
- ( ) No
  No
- ( ) Refused to answer
  Se negó a responder

Questions about Social Circle:

Do you have a relative or close friend (who you have not yet mentioned) who:

Preguntas sobre su Círculo Social:

¿Tiene un familiar o amigo cercano (que aún no ha mencionado) que:

29 **29. Had symptoms of COVID-19?**

Ha tenido síntomas de COVID-19?

- ( ) Yes
  Sí
- ( ) No
  No
- ( ) Refused to answer
  Se negó a responder

30. Was diagnosed with COVID-19?

Le diagnosticaron COVID-19?

- ( ) Yes
  Sí
- ( ) No
  No
- ( ) Refused to answer
  Se negó a responder

31. Was hospitalized with COVID-19?

Fue hospitalizado con COVID-19?

- ( ) Yes
  Sí
- ( ) No
  No
- ( ) Refused to answer
  Se negó a responder

32. Passed away/died from COVID-19?

Falleció / murió de COVID-19?

- ( ) Yes
  Sí
- ( ) No
  No

- ◯ Refused to answer
  Se negó a responder

↺

SECTION D: HOUSEHOLD CONDITIONS

Thank you so much for sharing information about your family during this difficult time. Now we're going to ask some questions about your housing and living situation.

Housing:

Muchas gracias por compartir información sobre su familia durante este momento difícil. Ahora vamos a hacer algunas preguntas sobre su vivienda y su situación de vida.

Alojamiento:

33. Where did you sleep most nights from March - August 2020?

I will read you a list of options and let me know if you agree with any of the following statements.

Dónde durmió la mayoría de las noches de Marzo a Agosto del 2020?

Le leeré una lista de opciones y me avisara si está de acuerdo con alguna de las siguientes afirmaciones.

- ☐ room, or apartment that you own
  Casa, habitación o apartamento que alquila
- ☐ [BRANCHING] 33.1- If yes, is it a New York City Housing Authority (or NYCHA) operated building? Yes/No/Not Sure/Refused to Answer
  Casa, habitación o apartamento que alquila En caso afirmativo, ¿es un edificio operado por la Autoridad de Vivienda de la Ciudad de Nueva York (o NYCHA)? Sí / No / No estoy seguro / Se negó a responder
- ☐ With a friend or relative
  Casa, habitación o apartamento de su propiedad
- ☐ In an emergency shelter, safe haven, or transitional housing
  En un refugio de emergencia, refugio seguro o vivienda de transición
- ☐ In an institution (jail, prison, long-term care facility, or nursing home)
  En una institución (cárcel, prisión, centro de cuidados a largo plazo o asilo de ancianos)
- ☐ Other (in a car, on the street)
  Otro (en un automóvil, en la calle)
- ☐ Refused to answer
  Se negó a responder

34. How many bedrooms were there in that home?

Cuántos dormitorios había en esa casa?

[▾]

35. Did you mostly stay in New York City during this

time, or did you change homes?

Se quedó mayormente en la ciudad de Nueva York durante este tiempo o cambió de hogar?

- Stayed in New York City
  Me alojé en la ciudad de Nueva York
- Changed homes
  Cambio de casa
- Refused to answer
  Se negó a responder

↺

The next few questions are about the home where you were living most nights during the same time period, between March 2020 - August 2020:

Las siguientes preguntas son sobre el hogar donde vivió la mayoría de las noches durante el mismo período de tiempo, entre marzo del 2020 y agosto del 2020:

36. How did {the child with asthma} spend the majority of {his/her/their} day?

I will read you a list of options and let me know if you agree with any of the following statements. You can pick more than one option.

[Note to Interviewer: read option and check all that apply]

Cómo pasó {el niño/a con asma} la mayor parte de su día? Le leeré una lista de opciones y me avisa si está de acuerdo con alguna de las siguientes opciones. Puede elegir más de una opción.

[Nota para el entrevistador: lea la opción y marque todas las que correspondan]

- at home with a parent
  en casa con un padre
- at home with a caregiver (grandma, aunt, older sibling, etc)
  en casa con un cuidador (abuela, tía, hermano mayor, etc.)
- outside of the home at day care
  fuera de casa en la guardería
- outside of the home at a day summer camp
  fuera de casa en un campamento de verano
- outside of the home with a family friend/relative
  fuera de casa con un amigo / pariente de la familia
- inside of the home at a virtual/on-line school or day summer camp
  dentro de la casa en una escuela virtual / en línea o campamento de verano
- other: (please specify) _____
  otro: (especifique)
- Refused to answer
  Se negó a responder

37.Have you seen cockroaches inside your home?

Has visto cucarachas dentro de tu casa?

- Yes
  Sí
- No
  No
- Don't Know/Not Sure
  No sé/No estoy seguro
- Refused to answer
  Se negó a responder

↺

38.Have you seen evidence of mice or rats inside your home?

- Yes
  Sí

Ha visto evidencia de ratones o ratas dentro de su casa?

- No
  No
- Don't Know
  No sé
- Refused to answer
  Se negó a responder

39.Have you seen or smelled mold/mildew in your home?

Ha visto u olido moho / hongos en su casa?

- Yes
  Sí
- No
  No
- Don't Know
  No sé
- Refused to answer
  Se negó a responder

40. Is there evidence of water damage in your home?

Hay evidencia de daños por agua en su hogar?

- Yes
  Sí
- No
  No
- Don't Know
  No sé
- Refused to answer
  Se negó a responder

41.Is there wall-to-wall carpet in your child's bedroom?

Hay alfombra de pared a pared (piso completo) en el dormitorio de su hijo/a?

- Yes
  Sí
- No
  No
- Don't Know
  No sé
- Refused to answer
  Se negó a responder

42.Do you have any furry pets?

Tienes mascotas peludas? Ejemplos: perros, gatos

- Yes
  Sí
- No
  No
- Don't Know
  No sé
- Refused to answer
  Se negó a responder

43.How often does anyone smoke inside your home?

Con qué frecuencia fuma alguien dentro de su casa?

- Daily
  Diariamente
- Weekly
  Semanal
- Monthly
  Mensual
- Less than monthly
- Never
- Don't know
- Refused to answer
  Se negó a responder

44.Are you worried about losing your housing?

Le preocupa perder su vivienda?

- Yes [Note to Interviewer: If answers yes, provide information in follow up email regarding housing resources
- No
  No
- Don't know
  No se
- Refused to Answer
  Se negó a responder

SECTION E: SOCIAL DETERMINANTS OF HEALTH

Thank you so much for sharing this information with us. I am now going to ask about other things that may have affected your family during the pandemic

SECCIÓN E: DETERMINANTES SOCIALES DE LA SALUD

Muchas gracias por compartir esta información con nosotros. Ahora voy a preguntar sobre otras cosas que pudieron haber afectado a su familia durante la pandemia.

45.During this same time period (between March 2020 - August 2020), how did you travel to do the things you need to do, such as going to work, appointments or shopping? (Please check all that apply)

I will read you a list of options and let me know if you agree with any of the following statements. You can pick more than one option.

Durante este mismo período de tiempo (entre marzo del 2020 y agosto del 2020), ¿cómo viajó para hacer las cosas que necesita hacer, como ir al trabajo, citas o compras? (Por favor marque todos los que apliquen)

Le leeré una lista de opciones y me avisara si está de acuerdo con alguna de las siguientes afirmaciones. Puede elegir más de una opción

- Public transportation (Check all that apply: bus, subway, train {such as LIRR, Metro North, NJ Transit},or ferry)
  Transporte público (marque todo lo que corresponda: autobús, metro, tren {como LIRR, Metro-North, NJ Transit} o ferry)
- Walk
  Caminar
- Bike
  Bicicleta
- Own car
  Coche propio
- Rental car
  Coche de alquiler
- Taxi car
  Taxi

- [ ] Car service (like Uber or Lyft
  Servicio de automóvil (como Uber o Lyft)
- [ ] I don't need to travel/I don't leave my home
  No necesito viajar / no salgo de mi casa
- [ ] Refused to answer
  Se negó a responder

Food Insecurity/Access:

46. Since the pandemic started, how much did you worry that your food would run out before you got money to buy more?

Inseguridad alimentaria / acceso:

Desde que comenzó la pandemia, ¿cuánto le preocupaba que se le acabara la comida antes de tener dinero para comprar más?

- [ ] Never
  Nunca
- [ ] Sometimes
  A veces
- [ ] Often
  A menudo
- [ ] Refused to answer
  Se negó a responder

We are now going to ask questions about how you learned about COVID-19 and your understanding of how to stay healthy and your opinion about the response.

Ahora vamos a hacer preguntas sobre cómo se enteró del COVID-19 y su comprensión de cómo mantenerse saludable y su opinión sobre la respuesta.

47. What are the main ways you get information about the pandemic?

I will read you a list of options and let me know if you agree with any of the following statements. You can pick more than one option.

Cuáles son las principales formas de obtener información sobre la pandemia?

Le leeré una lista de opciones y me avisaré si está de acuerdo con cualquiera de las siguientes declaraciones. Puedes elegir más de una opción.

[Nota para el entrevistador - marque todos los que correspondan]

- [ ] Government health websites (like the CDC or Department of Health)
  Pagina de salud del gobierno (como el CDC o el Departamento de Salud)
- [ ] Searching for information online (like Google)
  para obtener información por internet(como Google)
- [ ] Social media (like Facebook, Instagram, and Twitter)
  redes sociales (como Facebook, Instagram y Twitter)
- [ ] Television news or radio
  Noticias de televisión o radio
- [ ] Newspaper, magazines
  periódico, revistas
- [ ] Family/Friends
  Familia/Amigos
- [ ] Health care providers, like doctors
  Proveedores de Salud, como los doctores
- [ ] Community or faith leaders
  líderes de la comunidad o líderes de fe
- [ ] Other (please specify): _
  Otro (por favor especifica):
- [ ] Refused to answer
  Se negó a responder

[Interviewer note - check all that apply]

48. How often do you get information about the pandemic?

Con qué frecuencia obtiene información sobre la pandemia?

- Less than once a month
  Menos de una vez al mes
- Several times a month
  Varias veces al mes
- Several times a week
  Varias veces a la semana
- Once a day
  Una vez al día
- 2-10 times a day
  2-10 veces al día
- More than 10 times a day
  Más de 10 veces al día
- Refused to answer
  Se negó a responder

49. How informed would you say you are about the current pandemic?

Qué tan informado diría que usted está sobre la pandemia?

- Not very informed
  No tan informado
- Somewhat informed
  Un poco informado
- Very informed
  Muy informado
- Refused to answer
  Se negó a responder

50. I have or I will get the flu vaccine this year

Yo ya me puse o me voy a poner la vacuna del Flu este año ?

51. If a COVID-19 vaccine becomes available and is recommended for me, I would get it

Si la vacuna de Covid-19, llega a estar disponible y es recommendable para mi, me la pondre

- Strongly Disagree
  Muy en desacuerdo
- Disagree
  Desacuerdo
- Neutral
  Neutral
- Agree
  De acuerdo
- Strongly Agree
  Muy de Acuerdo
- Don't Know
  No sé

52. Does information about COVID-19 ever make you feel anxious/overwhelmed?

- Yes - Often

La información sobre COVID-19 te hace sentir ansioso/abrumado?

Sí - A menudo
- ◯ Yes - Sometimes
  Sí - A veces
- ◯ No
  No
- ◯ Refused to answer
  Se negó a responder

53. Can someone with COVID-19 infect others without feeling sick or without showing any symptoms?

Puede alguien con COVID-19 infectar a otras personas sin sentirse enfermo o sin mostrar ningún síntoma?

- ◯ Yes
  Sí
- ◯ No
  No
- ◯ Refused to answer
  Se negó a responder

54. How does COVID-19 spread? (Select all that apply)

I will read you a list of options and let me know if you agree with any of the following statements. You can pick more than one option.

Cómo se propaga COVID-19? (Seleccione todos los que correspondan)

Le leeré una lista de opciones y me avisara si está de acuerdo con cualquiera de las siguientes declaraciones. Puede elegir más de una opción.

- ☐ When in close contact with an infected person (within about 6 feet).
  Cuando está en contacto cercano con una persona infectado (dentro de unos 6 pies).
- ☐ Through respiratory droplets produced when an infected person coughs or sneezes
  A través de gotas respiratorias producidas cuando una persona infectada tose o estornuda
- ☐ Touch a contaminated surface and then touching your eyes, nose or mouth.
  Toca una superficie contaminada y luego toque los ojos, la nariz o la boca.
- ☐ Though unprotected sex.
  A traves el sexo sin protección.
- ☐ The virus is a hoax.
  El virus es un engaño.
- ☐ Refused to answer
  Se negó a responder

55.How likely do you think you are to get sick from COVID-19 in the next month?

Score between 0 and 10 (drop down 0 not getting sick and 10 very likely getting sick, Refused to answer)

Qué tan probable cree que usted se enfermará de COVID-19 en el próximo mes? Puntuación entre 0 y 10 (abajo 0 no enfermarse y 10 muy probable que se enferme, se negó a responder)

56. Take 100 randomly selected people (not including you) who live in your zip code. How many, do you think, will get sick from COVID-19 in the next month?

____ (drop down 0 not getting sick and 100 very likely getting sick, Refused to answer)

Tome 100 personas seleccionadas al azar (sin incluir a usted) que viven en su código postal. ¿Cuántos, crees, que se enfermarán de COVID-19 en el próximo mes? ____ (baja 0 no enfermarse y 100 muy probablemente enfermarse, Se negó a responder)

57. Take 100 randomly selected people (not including you) who live in your zip code. How many, do you think, have already gotten sick from COVID-19? ____ (drop down 0 not getting sick and 100 very likely getting sick, Refused to answer)

Tome 100 personas seleccionadas al azar (sin incluir a usted) que viven en su código postal. ¿Cuántos crees que ya se han enfermado de COVID-19? ____ (baja 0 no enfermarse y 100 muy probable que se enfermen, se negó a responder)

58. Do you feel that the government's policies help keep you safe from the virus?

Cree que las políticas del gobierno ayudan a mantenerle a salvo del virus?

- Yes
  Sí
- No
  No
- Don't Know
  No sé
- Refused to answer
  Se negó a responder

Social Distancing/Hygiene:

59.0 The following statements ask about behaviors to stop the spread of

COVID-19. For each statement, please indicate how often you do these activities. The choices are not at all, some of the time, much of the time or all of the time:

Distanciamiento Social/Higiene Social:

Las siguientes declaraciones preguntan acerca de los comportamientos para detener la propagación de COVID-19. Para cada declaración, indique la frecuencia con la que realiza estas actividades. Las opciones no son en absoluto, parte del tiempo, la mayor parte del tiempo o todo el tiempo:

	Not at all no son en absoluto	Some of the time parte del tiempo	Most of the time la mayor parte del tiempo	All of the time Todo el Tiempo
**59.1 Use an alcohol-based hand sanitizer or wash your hands often with soap and water Use desinfectante de base de alcohol o lavarse las manos con frecuencia con agua y jabón**	○	○	○	○
	○	○	○	○

59.2 Clean surfaces in your home like doorknobs, faucets, and counter tops
Limpie las superficies de su hogar Como las chapas de la puerta, grifos y Encimeras

59.3. Wear a face mask or cover your face with a scarf, bandana, etc. when in public places
Use una máscara o cubierta facial como una bufanda, pañuelo, etc. cuando en lugares públicos

59.4 Stay at home, except to get food or medicine or go to work
Se queda en casa, excepto para comida o medicinas o ir a trabajar

59.5 Reduce or avoid using healthcare facilities such as hospitalsor clinics
Reducir o evitar el uso de lugares médica como hospitales o clínicas

59.6 Avoid public transportation
Evitar el transporte público

59.7 Avoid overly crowded places and gatherings of more than 10 people
Evitar el lugar lleno de gente y reuniones de más de 10 personas

60. How difficult is it to engage in "social distancing" (keeping 6 feet away from people) when you are in public places?

Qué tan difícil es participar en el "distanciamiento social" (mantenerse a 6 pies de distancia de las personas) cuando estás en lugares públicos?

- Very difficult
  Muy difícil
- Difficult
  Difícil
- Easy
  Fácil
- Very easy
  Muy Fácil
- Refused to answer
  Se negó a responder

We're almost done! We just have a few more questions....

SECTION G: DEMOGRAPHICS and EMPLOYMENT

Ya casi terminamos! Sólo tenemos algunas preguntas más....

SECCIÓN G: DEMOGRAPHICS y EMPLEO

61. What was the sex of {your child with asthma} at birth?

Cuál fue el sexo de su hijo/a con asma al nacer?

- Male
  Masculino
- Female

Mujer
- Intersex
  Intersexual
- Prefer not to say
  Prefiere no decir
- Refused to answer
  Se negó a responder

62. What is the race/ethnicity of {your child with asthma]}? (Please check all that apply)

Cuál es la raza/etnicidad de su hijo/a con asma? (Por favor marque todos los que correspondan) Hispano o Latino/Latinox

- Hispanic or Latino/Latinx
  Hispano o Latino/Latinox
- Black or African American
  Negro o afroamericano
- White
  Blanco
- Asian
  Asiático
- Native Hawaiian or Pacific Islander
  Nativo hawaiano o isleño del Pacífico
- American Indian or Alaskan Native
  Indio americano o nativo de Alaska
- Other race (please specify):
  Otra raza (especificar):
- Refused to answer
  Se negó a responder

63. What is the highest level of school that you finished?

Cuál es el nivel más alto de escuela que terminó?

- Did not attend school
  No asistió a la escuela
- Less than 8th grade
  Menos de grada 8
- Some high school degree
  Algunos títulos de bachillerato
- High school degree or GED
  Diploma de escuela Secundaria o GED
- Some college
  Ir a la universidad
- Bachelor's degree
  Licenciatura
- Post graduate degree
  Posgrado
- Refused to answer
  Se negó a responder

64. Has your job been affected by COVID-19 (Coronavirus) in any of the following ways?

I will read you a list of options and let me know if you

- Moved to remote work
  Se ha trasladado al trabajo remoto
- Lost job permanently

agree with any of the following statements. You can pick more than one option.

Su trabajo ha sido afectado por COVID-19 (Coronavirus) de alguna de las siguientes maneras??

Le leeré una lista de opciones y me avisa si está de acuerdo con cualquiera de las siguientes declaraciones. Puede elegir más de una opción.

[Nota del entrevistador: marca todos lo que aplica]

Trabajo perdido permanentemente
- ☐ Lost job temporarily
  Trabajo perdido temporalmente
- ☐ Hours have been reduced
  Horas se han reducido
- ☐ Hours have increased
  Horas han aumentado
- ☐ Job was designated as an essential service
  Su trabajo fue designado como un servicio esencial
- ☐ Your job puts you at increased risk of being infected by COVID-19 (Coronavirus)
  Su trabajo le pone en mayor riesgo de ser infectado por COVID-19 (Coronavirus)
- ☐ You had to lay off employees
  Tuvo que despedir a sus empleados
- ☐ None of these apply
  Ninguno de estos se aplica
- ☐ Job has not been affected by COVID-19
  Job no se ha visto afectado por COVID-19
- ☐ Refused to answer
  Se negó a responder

[Interviewer note - check all that apply]

[BRANCHING if participant works outside of home] 64.1 Does your job provide you with PPE (personal protective equipment)?

si el participante trabaja fuera de casa] 64.1 ¿Su trabajo le proporciona EPP (equipo de protección personal)?

- ◯ Yes
  Si
- ◯ No
  No
- ◯ I bring my own
- ◯ Not sure
- ◯ Refused to answer
  Se negó a responder

↺

---

65. Has your spouse/partner's job been affected by COVID-19 (Coronavirus) in any of the following ways? (Mark all that apply)

I will read you a list of options and let me know if you agree with any of the following statements. You can pick more than one option.

El trabajo de su esposo o esposa/pareja se ha visto afectado por COVID-19(Corona virus) de alguna de las siguientes maneras? (Marque todo lo que corresponda)

Le leeré una lista de opciones y me avisa si está de acuerdo con cualquiera de las siguientes declaraciones. Puede elegir más de una opción.

- ☐ Not applicable - I do not have a spouse/partner
  No aplicable- No tengo esposo o esposa/pareja
- ☐ Your spouse/partner moved to remote work
  Su Esposo o Esposa/pareja se trasladó a un trabajo remoto
- ☐ Your spouse/partner lost his/her job permanently
  Su Esposo o Esposa/pareja perdió su trabajo de forma permanente
- ☐ Your spouse/partner lost his/her job temporarily
  Su Esposo o Esposa/pareja perdió su

[Nota del entrevistador: marque todo lo que corresponda]

trabajo temporalmente

- [ ] Your spouse/partner had his/her hours reduced
Su Esposo o Esposa/pareja le redujeron sus horas
- [ ] Your spouse/partner's hours have increased
las horas de su Esposo o Esposa/pareja han aumentado
- [ ] Your spouse/partner's job was designated as an essential service
el trabajo de su Esposo o Esposa/pareja fue designado como un servicio esencial
- [ ] Your spouse/partner's job puts him/her at increased risk of being infected by COVID-19 (Coronavirus)
el trabajo de su Esposo o Esposa/pareja le pone en mayor riesgo de ser infectado por COVID-19 (Coronavirus)
- [ ] Your spouse/partner had to lay off employees
Su Esposo o Esposa/pareja tuvo que despedir a sus empleados
- [ ] None of these apply
Ninguno de estos se aplica
- [ ] Your spouse/partners job has not been affected by COVID-19
Su trabajo de Esposo o Esposa/pareja no se ha visto afectado por COVID-19
- [ ] Don't know
No se
- [ ] Refused to answer
Se negó a responder

[Interviewer note - check all that apply]

66. What was your household's total family income (before taxes) from all sources in the last year?

Cuál fue el ingreso familiar total de su hogar (antes de impuestos) de todas las fuentes en el último año?

- ( ) Less than $20,000 (or less than $1,666 per month)
Menos de $20,000 (o menos de $1,666 por mes)
- ( ) $20,000 to $39,999 (or $1,666 to $3,333 per month)
de$20,000 a $39,999 (o $1,666 a $3,333 por mes)
- ( ) $40,000 to $59,999 (or $3,333 to $4,999 per month)
de $40,000 a $59,999 (o $3,333 a $4,999 por mes)
- ( ) $60,000 to $79,999 (or $5,000 to $6,666 per month)
de $60,000 a $79,999 (o $5,000 a $6,666 por mes)
- ( ) $80,000 to $99,999 (or $6,666 to $8,333

per month)
de $80,000 a $99,999 (o $6,666 a $8,333 por mes)
- $100,000 to $139,999 (or $8,333 to $11,666 per month)
$100,000 a $139,999 (o $8,333 a $11,666 per mes)
- $140,000 or more (or $11,666 or more per month)
$140,000 o más (o $11,666 o más por mes)
- Don't know
No se
- Refused to answer
Se negó a responder

67. Has your family income changed during the pandemic:

Ha cambiado los ingresos de su familia durante la pandemia:

- Yes - we are earning more money
Sí - estamos ganando más dinero
- Yes - we are earning less money
Sí - estamos ganando menos dinero
- No - income has not changed
No - los ingresos no han cambiado
- Refused to answer
Se negó a responder

68. Insurance: What kind of health insurance does {your child with asthma} currently have?

Seguro: ¿Qué tipo de seguro de salud tiene actualmente? su hijo/a con asma?

- No insurance/sliding scale
Sin seguro
- Medicaid
Medicaid
- Private health insurance
Seguro médico privado
- Other insurance (please specify):
Otro seguro (especificar):
- Don't know
No se
- Cobra insurance
Seguro de cobra
- Refused to answer
Se negó a responder

69. Has {your child with asthma}'s medical insurance changed during the start of the pandemic?

¿Ha cambiado el seguro médico de su hijo/a con asma durante el inicio de la pandemia

- Yes
Sí
- No
No
- Don't Know
No sé
- Refused to answer

Se negó a responder

[BRANCHING] 69.1 If Yes:

Did you lose the health insurance that covers {your child with asthma} since the pandemic started?

En caso afirmativo:

o ¿Perdió el seguro de salud que cubre a su hijo/a con asma desde que comenzó la pandemia?

- Yes
  Sí
- No
  No
- Don't Know
  No sé
- Refused to answer
  Se negó a responder

70. [Comments] à "Comments: __________" or "Any other thoughts or experiences you want to share about the pandemic _________________?"

70. [Comentarios] - "Comentarios: __________" o "Cualquier otro pensamiento o experiencia que desee compartir sobre la pandemia __________?"

Expand

Thank you for taking the time to help us learn about healthcare during COVID-19. We appreciate you sharing your experiences with us. We would like to offer you a $40 gift card as an appreciation of your willingness to talk with us. Can I please have your email address to send the gift card? We will also be sending you information about resources that may be helpful to answer questions about asthma and environmental asthma triggers, as well as any resources that may be helpful to your family based on your survey responses.

Gracias por tomarse el tiempo para ayudarnos a aprender sobre la atención médica durante COVID-19. Agradecemos que comparta sus experiencias con nosotros. Nos gustaría ofrecerle una tarjeta de regalo de $40 como una apreciación de su disposición a hablar con nosotros. ¿Puedo tener su dirección de correo electrónico para enviar la tarjeta de regalo? También le enviaremos información sobre recursos que pueden ser útiles para responder preguntas sobre el asma y los desencadenantes del asma ambiental, así como todos los recursos que pueden ser útiles para su familia en función de las respuestas de su encuesta.

How would you like to receive your gift card:
By mail or coordinate a pickup at Mount Sinai, 17 east 102nd street or
LSA, 333 East 110th st?

- Mail
- Pick up Mount Sinai
- Pick up LSA

✔
